# Nitrogen and Hydrogen Adsorption by an Organic Microporous Crystal**

**DOI:** 10.1002/anie.200900234

**Published:** 2009-04-02

**Authors:** Kadhum J Msayib, David Book, Peter M Budd, Nhamo Chaukura, Kenneth D M Harris, Madeleine Helliwell, Steven Tedds, Allan Walton, John E Warren, Mingcan Xu, Neil B McKeown

**Affiliations:** School of Chemistry, Cardiff UniversityCardiff CF10 AT (UK) E-mail: mckeownnb@Cardiff.ac.uk Homepage: http://www.cardiff.ac.uk/chemy/staffinfo/mckeown/; School of Chemistry, University of ManchesterManchester, M13 9PL (UK); School of Metallurgy and Materials, College of Engineering and Physical Sciences, University of BirminghamBirmingham, B15 2TT (UK); CCLRC Daresbury LaboratoryDaresbury, Warrington, Cheshire, WA4 4AD (UK)

**Keywords:** adsorption, hydrogen storage, interconnectivity, microporous materials, organic zeolites

Inspired by the impressive properties of organic–inorganic hybrid microporous materials, such as metal–organic frameworks (MOFs),^1^ the preparation of purely organic microporous materials is currently of intense interest. Examples of recently developed organic microporous materials include the crystalline covalent organic frameworks (COFs),^2^ and amorphous polymers such as the versatile polymers of intrinsic microporosity (PIMs).^3^ In addition, an emerging area of interest is the study of crystals of low-molecular-mass organic molecules that can behave analogously to microporous materials by providing accessible internal surface areas for the reversible adsorption of small molecules. Ultimately, such crystals may provide alternative materials for a range of applications, including gas separations, capture, and storage.^4^

It has long been known that crystals derived from various organic compounds can act as hosts for solvent or gas molecules.^5^ These organic clathrates are usually unstable toward the removal of the included species, although some allow the exchange of one type of guest for another and a few survive the evacuation of included solvent.^6^ Such crystals are sometimes termed “organic zeolites”,^7^, ^8^ although their structures and properties have little in common with those of the zeolites.^9^ In particular, rapid and reversible gas adsorption is a difficult challenge for microporous organic crystals.^10^ More recently, several organic crystals have exhibited robust ‘permanent’ microporosity by reversible gas adsorption. The example that has been studied the most is the microporous crystal of tris(*o*-phenylenedioxy)cyclotriphosphazene (TPP),^11^–^13^ for which the adsorption of significant quantities of CO_2_,^14^ N_2_,^12^ Xe,^11^, ^12^ and CH_4_^14^ into its 4.5 Å diameter linear channels has been successfully demonstrated. Recent studies have also described gas adsorption within evacuated crystals of cuburbit[6]uril (acetylene and N_2_),^15^ *p-tert*-butylcalix[4]dihydroquinone (CO_2_ and N_2_),^16^, ^17^ and dipeptides (CO_2_, CH_4_, and H_2_).^18^, ^19^

In contrast to MOFs,1a COFs,2b and some hydrogen-bonded crystals,^20^ whose ordered structures can be engineered with some degree of certainty,^21^ the packing of small molecules within organic crystals can be difficult to predict. Hence, further examples of microporous organic crystals are more likely to be discovered rather than prepared by following a rational design. Furthermore, we considered it highly likely that potentially microporous organic crystals have been previously characterized by X-ray diffraction (XRD) but not recognized as such, simply because the sole objective of the analysis was the confirmation of the molecular structure of the organic compound. Hence, we set out to discover unrecognized microporous crystals by searching through the many crystal structures (more than 4.5×10^5^) held within the Cambridge Structural Database (CSD). Such an approach has been likened previously to “sieving the desert”,^8^ however, we narrowed the search criteria to identify candidates that might possess enhanced microporosity over existing examples of microporous crystals. The initial structure selection was based upon the following criteria: 1) a calculated density of less than 0.9 g cm^−3^ (the lowest density of any established microporous organic crystal is 0.96 g cm^−3^ for *p-tert*-butylcalix[4]dihydroquinone on removal of included water),^16^, ^17^ 2) the crystal should be composed predominantly of rigid aromatic molecules to aid stability, and 3) the apparent porosity should exist as small micropores (diameter<10 Å) to be suitable for strong gas adsorption through multiwall interactions.

Of a number of potential candidates identified by the search (see the Experimental Section), the crystal structure (CCDC 179393; BALNIM) formed by 3,3′,4,4′-tetra(trimethylsilylethynyl)biphenyl **1** (Figure 1) looked particularly intriguing because of its striking resemblance to that of a typical zeolite. The crystal is of cubic symmetry and belongs to the rare space group 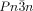,^22^ with a large unit cell (*a*=29.575(2) Å) that contains 24 molecules of **1** and a reported density of only 0.830 g cm^−3^. The crystal appears to possess channels of 4.0 Å minimum diameter, which interconnect voids of 11 Å maximum diameter to give a pore structure that is superficially similar to that of zeolite A (Figure 2).^9^ The structure also resembles the bicontinuous Schwarz P minimal surface that occurs in a number of colloidal and biological systems.^23^

**Figure 1:**
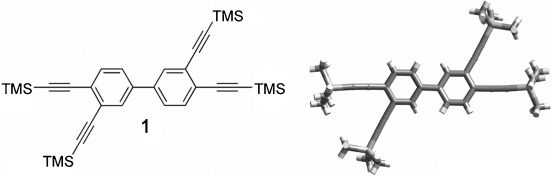
The molecular structure of **1** (TMS=trimethylsilyl) and its structure derived from X-ray single-crystal analysis.

**Figure 2:**
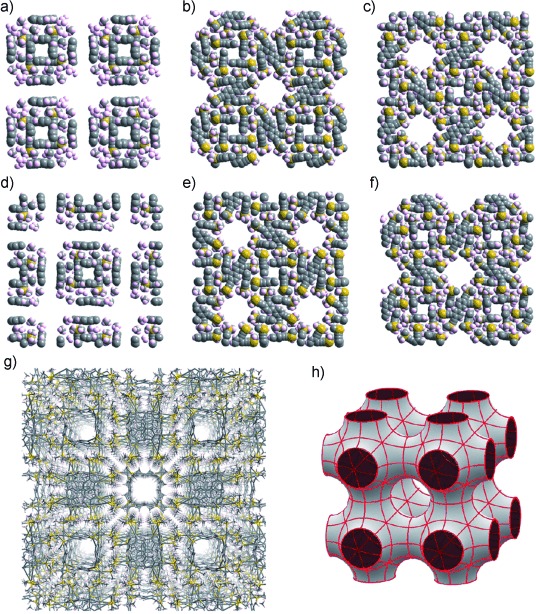
Depth profiling of the crystal structure of **1** showing 5 Å slices at a) 0–5 Å, b) 5–10 Å, c) 10–15 Å, d) 15–20 Å, e) 20–25 Å, and f) 25–30 Å depth through 2×2 unit cells depicted as space-filling models, which illustrates the bicontinuous network of open channels of 4 Å minimum diameter that connect voids of 11 Å maximum diameter. g) A perspective view of the crystal (2×2×2 unit cells). h) A cartoon representation of the Schwarz P minimal surface, which has the same topological features as the micropore structure of the crystal.

A sample of **1** was remade by following the straightforward method previously described.^24^ Colorless cubic crystals formed readily by evaporation of solvent from a concentrated hexane solution. A single-crystal XRD study confirmed the previously obtained structure deposited in the CSD. However, an electron count of 424 per unit cell revealed the presence of nonresolved molecules within the interconnected channels. Thermogravimetric analysis (TGA) of freshly prepared crystals indicates a loss of mass of 7.0 % up to the melting point of the compound (135 °C). The ^1^H NMR spectra of a solution of freshly prepared crystals in CDCl_3_ confirmed that the included solvent was hexane and that it was present at a ratio of one molecule of hexane for two molecules of **1**. The hexane can be readily removed from the crystals, without changing their appearance, by heating at 60 °C for several hours at 1 mbar, as confirmed by TGA and the ^1^H NMR spectrum of a solution of the sample in CDCl_3_. High-resolution solid-state ^13^C NMR spectroscopy also showed no evidence of residual hexane within the crystals. A single-crystal XRD analysis showed that the structure is retained after the evacuation of hexane. However, there is a significant reduction in the electron count within the channels to only 83 electrons per unit cell, which we assign to molecules adsorbed from the atmosphere during crystal manipulation.

The unit cell of the evacuated crystal is smaller (*a*=29.238(2) Å) than that recorded for the original structure deposited in the CSD (*a*=29.575(2) Å), although this difference could have been attributed to the different temperatures at which the XRD studies were carried out, that is, 150 K and 173 K, respectively. However, a direct comparison of a hexane-containing crystal and an evacuated crystal performed on the same diffractometer and at the same temperature (100 K) confirmed that the unit cell of the evacuated crystal is significantly smaller (*a*=29.047(4) Å) than that of the hexane-containing crystal (*a*=29.233(2) Å), which represents a 2 % reduction of the initial volume of the crystal on removal of hexane. Based on the estimate of a linear thermal expansion of the lattice of 0.19 Å K^−1^. We conclude that the structure previously deposited in the CSD was that of a hexane-containing crystal.

An evacuated crystalline sample of **1** shows a very significant degree of nitrogen adsorption at 77 K, especially at low relative pressure (3.3 mmol at *P*/*P*_0_=0.1), which is consistent with microporosity. At first glance, and by following the general classification of adsorption isotherms, the general shape of the adsorption isotherm appears to be a simple Type I isotherm (Figure 3).^25^ A Brunauer–Emmett–Teller (BET) surface area of 278 m^2^ g^−1^ and a micropore volume of 0.16 mL g^−1^ can be calculated from these data. The total amount of nitrogen adsorption of 4.4 mmol g^−1^ at *P*/*P*_0_=1 represents the greatest amount adsorbed by a crystalline material composed of a low-molecular-mass organic compound reported to date. The fundamental parameters derived from the nitrogen adsorption data of the crystal from **1** with those of the previously reported examples are compared in Table 1. A closer look at the low-pressure region of the nitrogen adsorption isotherm reveals two distinct inflections (Figure 3, inset a), which can be correlated with the filling of the two different micropore structures within the crystal: firstly the channels of 4 Å diameter, and secondly, the 11 Å voids. Analysis of these low-pressure nitrogen adsorption data by using the Horvath–Kawazoe method^26^ gives a bimodal pore size distribution. The well-defined peak centered at a diameter of 11 Å is consistent with the maximum size of the voids within the crystal (Figure 3, inset b). The channels between these voids are too narrow to be modeled because of the limitations of this technique, but they are represented by the distorted peak at 6 Å.

**Figure 3:**
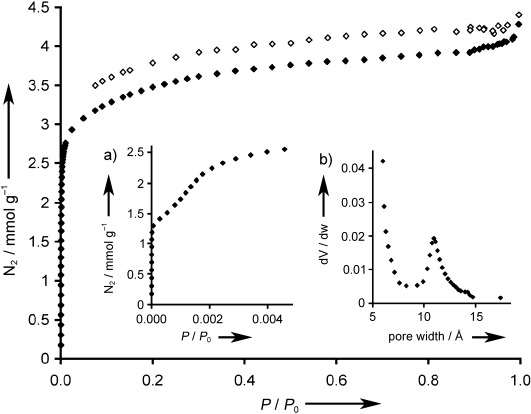
The nitrogen adsorption isotherm for crystals of **1** at 77 K (⧫ adsorption; ◊ desorption). The insets show a) an expansion of the low-pressure region of the isotherm showing the two distinct inflection points that correspond to nitrogen filling of the 4 Å channels and 11 Å voids and b) the pore size distribution calculated from the low-pressure adsorption data by using the Horvath–Kawazoe method.

**Table 1:** A comparison of nitrogen adsorption data at 77 K for microporous crystals of organic compounds

	*V* (N_2_)^[a]^ [mmol g^−1^]	Surface area (BET) [m^2^ g^−1^]	*V_p_*^[b]^ [ml g^−1^]	Ref.
**1**	4.4	278	0.16	–
TPP^[c]^	2.5	240^[f]^	0.09	12
CalixDHQ^[d]^	4.0	230	0.14	16
CB[6]^[e]^	3.8	210	0.13	15

[a] Amount of nitrogen adsorbed at *P*/*P*_0_=1.0; [b] micropore volume calculated from the amount of nitrogen adsorbed at *P*/*P*_0_=1.0; [c] tris(*o*-phenylenedioxy)cyclotriphosphazene; [d] *p-tert*-butylcalix[4]dihydroquinone; [e] cuburbit[6]uril; [f] calculated by using the Langmuir model.

The crystals of **1** also adsorb a significant amount of hydrogen at 77 K with 0.80 % uptake by mass (3.9 mmol g^−1^) at 10 bar (Figure 4). The only other published analysis of hydrogen adsorption within a microporous crystal of an organic compound is a very recent study based on dipeptide crystals,^18^ which demonstrates a maximum uptake of 0.45 % by mass (2.1 mmol g^−1^) at 10 bar and 77 K. The hydrogen uptake within the crystals of **1** is consistent with the general correlation between hydrogen adsorption and apparent surface area (or micropore volume) for a diverse range of microporous materials (e.g. MOFs, activated carbon-containing materials, and organic polymers) previously assessed as potential hydrogen-storage materials.^27^

**Figure 4:**
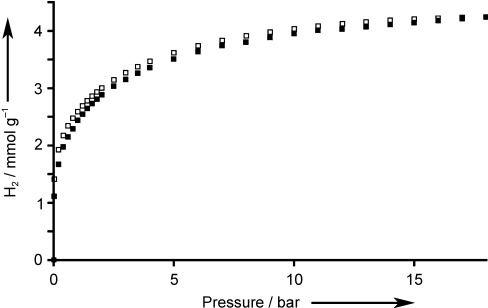
The hydrogen adsorption isotherm for crystals of **1** at 77 K. (▪ adsorption; □ desorption).

At first glance, the stability of the microporous crystals of **1** is surprising as there are no strong hydrogen-bond-donor or acceptor groups of the type that help to reinforce the microporous crystals formed by *p-tert*-butylcalix[4]dihydroquinone,^16^, ^17^ cuburbit[6]uril,^15^ or dipeptides.^18^ Such groups are also absent in TPP, however, the microporous crystal of TPP is unstable relative to its nonporous form so that care must be taken during its preparation to obtain microporosity by restricting the size of the crystals.^12^, ^13^ One feature that appears to contribute to the structural stability of the crystal is the neat self-assembly of four molecules of **1** into a hollow tube with a square-shaped cross-section (Figure 5). The four molecules are entwined to maximize the self-complementary CH–π interactions^28^ between the hydrogen atoms on the 2,2′,6,6′-positions of the biphenyl cores and the nearest acetylenic carbon atoms on adjacent molecules (*d*(PhH⋅⋅⋅C)<2.90 Å), so that each molecule is held in place by a total of eight such weak interactions (Figure 5 b). The unit cell of the crystal is composed of six such macrocyclic tetramers. Each tetramer is packed side-by-side with four neighbors so that their fourfold axis of symmetry, which runs through the hollow centre of the tetramer, is perpendicular to that of the central tetramer. This arrangement produces the remarkable bicontinuous micropore structure of the crystal (Figure 2). Porous solids that possess the Schwarz P minimal surface demonstrate optimal stress distribution,^29^ and this may help maintain structural integrity of the crystal during evacuation of included solvent. This stability has the practical advantage that there is no need to limit the crystal size during their preparation. Indeed, relatively large crystals (of average diameter=0.3 mm) were used both for the single-crystal XRD and gas adsorption studies.

**Figure 5:**
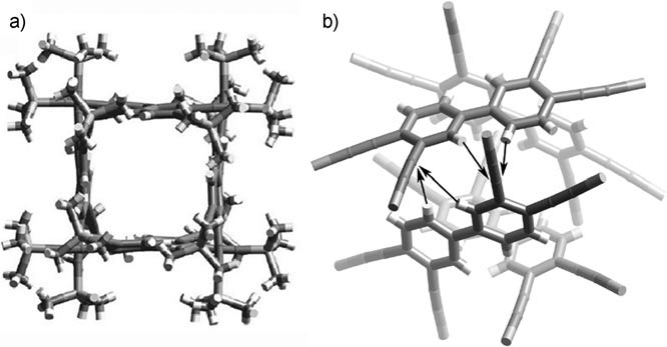
a) Face-on and b) edge-on views of the cyclic tetrameric assembly of **1** that is the basic structural unit of the molecular packing within the crystal. The arrows indicate the self-complementary CH–π interactions (C–H⋅⋅⋅C distance<2.90 Å) that stabilizes the structure. Note that the methyl groups in (b) are not shown for clarity.

Another attractive structural feature of the crystals of **1** is the three-dimensional interconnectivity of the void space, which means that, unlike the one-dimensional linear channels of the microporous crystals of TPP, dipeptides, and cuburbit- [6]uril, the adsorbate molecules can access the micropores from all facets of the crystal. This feature is shared by many zeolites, MOFs, and microporous *p-tert*-butylcalix[4]dihydroquinone-based crystals,^16^, ^17^ and is important as it allows multiple paths for the adsorbents to access each micropore and avoids potential reduction of adsorption because of pore-blocking. The three-dimensional interconnectivity of microporosity is expected to enhance the kinetics of adsorption in comparison with crystals of one-dimensional channels of a similar size. Although based on a very different length scale, modeling studies of macroporous structures have shown that the Schwarz P minimal surface represents the optimal structure for permeability.^30^

The discovery of the microporous crystal of **1** by scanning the CSD under a set of well-defined search criteria justifies the reinvestigation of existing crystal structures in order to expand the small number of known examples of this class of material. A rich seam of promising candidate structures with apparent microporosity has been identified for future analysis. However, it should be noted that the search criteria used in this study would not identify every potential microporous organic crystal. For example, XRD analysis of a clathrate often locates ordered solvent molecules within its structure that will often increase the density of the crystal to over 0.9 cm^−3^, even though the evacuated crystal may be of much lower density. Similarly, the presence of highly disordered solvent, such as the hexane molecules in the crystals of **1**, is often accounted for by using software programs such as SQUEEZE. Nevertheless, is notable that, had this approach been used during the original analysis of the crystal of **1**, it would still have be identified in the present study.

## Experimental Section

CSD search methodology: The CSD database (Version 5.30; November 2008) was searched using the ConQuest interface by restricting the structures to a density of less than 0.9 g cm^−3^. From the initial 519 hits, 218 structures were rejected as they are composed predominantly of saturated hydrocarbon or other components (e.g., B, Li), which provide low density but nonporous crystals. A further 122 were rejected because they are composed of inorganic or inorganic–organic building units (including 28 MOF or MOF-like structures). Of the remaining organic structures, 13 were eliminated as they possess pores of diameter greater than 10 Å (including 5 COF structures). A further 31 structures proved to have questionable values for density or a dubious structure as identified in the CSD or by using the CheckCIF online program and 27 structures could not be evaluated because of insufficient data (i.e., no available cif). The following structures satisfy the search criteria and are potential candidates for purely organic microporous crystals: ABINOP,^31^ BALNIM,^24^ EFALEC,^32^ FAKTIV,^33^ GIPTOO,^34^ KETYEO,^35^ NASQAA,^36^ PETREM,^37^ RERNEI,^38^ SULDUY^39^ TOZZIR,^40^ WAVJAE,^41^ XICRUW,^42^ XOPYEG,^43^ and YUPTIM.^44^ In addition, the following metal-containing structures are candidates as microporous molecular crystals: ADIYIV,^45^ FOSTEM,^46^ FOSTEM10,^47^ GOBSUL,^47^ IKANOX,^48^ KISYIV,^49^ PICKAN,^50^ and TIKFIC.^51^

Synthesis and crystal preparation: The preparation of **1** was as previously described.^24^ Following purification by column chromatography, the crystals were prepared by evaporation of a hexane solution. Removal of included hexane was achieved by heating the crystals at 60 °C in a vacuum oven for 12 h.

XRD data for the evacuated crystals of **1**: Data were collected at 150 K using synchrotron radiation at Daresbury SRS, UK (Station 9.8), on a Bruker APEXII CCD diffractometer (*λ*=0.69390 Å) and the structure was solved by direct methods. All calculations were carried out by using the SHELX-97 package. Crystal size 0.30×0.25×0.25 mm; cubic; space group 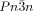, *a*=29.238(2) Å; *V*=24 995(3) Å^3^; *Z*=24; *Dx*=0.859 g cm^−3^, *μ*=0.197 mm^−1^; 3000 reflections measured; 3000 unique reflections (*R*_int_=0.0000); 2198 reflections with *I>2σ(I)*; *R*=0.0638 and w*R2*=0.1925 (observed data); *R*=0.0837 and w*R2*=0.2079 (all data). CCDC 713074 contains the supplementary crystallographic data for this paper. These data can be obtained free of charge from The Cambridge Crystallographic Data Centre via http://www.ccdc.cam.ac.uk/data_request/cif.

Gas adsorption studies: Volumetric nitrogen sorption studies were undertaken using a Micromeritics Instrument Corporation (Norcross, Georgia, USA) ASAP 2020 system. Before sorption analysis, the sample was subjected to the degas vacuum system under ultrahigh vacuum (10^−9^ bar) at a temperature of 60 °C for 8 h. The sample was back-filled with nitrogen and transferred to the analysis system. The sample was then again degassed under ultrahigh vacuum (10^−9^ bar) at a temperature of 50 °C for a period of 16 h, and kept at ultrahigh vacuum until analysis. Sorption analysis was carried out at 77 K. Helium was used for the freespace determination after sorption analysis, both at ambient temperature and at 77 K. Apparent surface areas were calculated from nitrogen adsorption data by multipoint BET analysis. Apparent micropore distributions were calculated from nitrogen adsorption data by the Horvath–Kawazoe method, assuming a slit–pore geometry and the original H–K carbon–graphite interaction potential. Gravimetric hydrogen sorption studies were undertaken at 77 K using a Hiden Isochema (Warrington, England) Intelligent Gravimetric Analyser (IGA). Before sorption analysis, the sample was degassed under ultra high vacuum (10^−9^ bar) at a temperature of 60 °C for a period of at least 6 h. Measured masses were corrected for buoyancy. The density values for buoyancy corrections (0.95 g mL^−1^) was obtained by helium pycnometry using a Micromeritics AccuPyc II 1340 System.
